# Decoding of four movement directions using hybrid NIRS-EEG brain-computer interface

**DOI:** 10.3389/fnhum.2014.00244

**Published:** 2014-04-28

**Authors:** M. Jawad Khan, Melissa Jiyoun Hong, Keum-Shik Hong

**Affiliations:** ^1^Department of Cogno-Mechatronics Engineering, Pusan National UniversityBusan, Republic of Korea; ^2^Department of Education Policy and Social Analysis, Columbia UniversityNew York, NY, USA; ^3^School of Mechanical Engineering, Pusan National UniversityBusan, Republic of Korea

**Keywords:** electroencephaelography, near-infrared spectroscopy, hybrid brain-computer interface, motor execution, arithmetic mental task, linear discriminant analysis

## Abstract

The hybrid brain-computer interface (BCI)'s multimodal technology enables precision brain-signal classification that can be used in the formulation of control commands. In the present study, an experimental hybrid near-infrared spectroscopy-electroencephalography (NIRS-EEG) technique was used to extract and decode four different types of brain signals. The NIRS setup was positioned over the prefrontal brain region, and the EEG over the left and right motor cortex regions. Twelve subjects participating in the experiment were shown four direction symbols, namely, “forward,” “backward,” “left,” and “right.” The control commands for forward and backward movement were estimated by performing arithmetic mental tasks related to oxy-hemoglobin (HbO) changes. The left and right directions commands were associated with right and left hand tapping, respectively. The high classification accuracies achieved showed that the four different control signals can be accurately estimated using the hybrid NIRS-EEG technology.

## Introduction

Brain-computer interface (BCI) is a methodology that correlates brain activities with external devices. The recent research and trend have demonstrated the enormous potential of the BCI approach (Matthews et al., 2008; Nicolas-Alonso and Gomez-Gil, 2012; Ortiz-Rosario and Adeli, 2013). The material advances in the cutting-edge technology, moreover, has reduced the cost of BCI equipment. The BCI domain comprehends both invasive and non-invasive methods. Invasive methods such as electrical-corticography (ECoG), though showing promising signal-acquisition results, are not recommended, as they entail very significant risks. Non-invasive methods are much safer alternatives in this regard (Min et al., 2010).

The major non-invasive modalities include electroencephalography (EEG), magneto encephalography (MEG), functional magnetic resonance imaging (fMRI), and functional near-infrared spectroscopy (fNIRS). Each has its own strengths and limitations; the selection of one over another for brain-imaging applications will rely on the cost of the equipment as well as the spatial and temporal resolution required for the given objective (Min et al., 2010).

EEG is a medical imaging technique that gauges brain activity by measuring, via metal electrodes positioned on the scalp, the voltage fluctuations on the scalp resulting from neurons' action potentials (Niedermeyer and Lopes da Silva, 1999; Rehan and Hong, 2012). The drawback of EEG is the poor spatial resolution that does not allow an accurate localization, that is, identification of the brain source signal (Ball et al., 2009).

NIRS is another non-invasive brain-imaging technique that alternatively utilizes the near-infrared (NIR) spectrum of light (wavelength 600–1000 nm) to measure the hemodynamic response represented by oxygenated hemoglobin (HbO), deoxygenated hemoglobin (HbR), cytochrome oxidase (CytOx) and water (H_2_O) concentration changes (Nagdyman et al., 2003; Irani et al., 2007; Bhutta et al., 2014). In most analyses, two hemodynamic variations due to brain activites are focused: increased oxygenation (resulting from the increased neural activity) and decreased deoxygenation (Matsuyama et al., 2009). Increased oxygen consumption in the course of performing increasingly difficult mental tasks has been demonstrated (Verner et al., 2013). fNIRS has also shown the ability to detect the fast optical response (Hu et al., 2011), however the hemodynamic response is mostly used for analysis.

EEG offers good temporal resolution (~0.05 s) but poor spatial resolution (~10 mm), while fNIRS provides only moderate temporal resolution (~1 s) and also moderately better spatial resolution (~5 mm) (Nicolas-Alonso and Gomez-Gil, 2012). Another advantage of fNIRS to EEG is its robustness to noise (Waldert et al., 2012). The objective of a hybrid BCI (Pfurtscheller et al., 2010) is either to improve the classification accuracy or/and to generate more control commands than the case of a single modality. The reason why Fazli et al. (2012) could improve the classification accuracy by using a hybrid EEG and fNIRS configuration is that they used the union of two cases (i.e., detected by either EEG or fNIRS). This was possible because the window size for which the features are identified has been set to include both fNIRS and EEG data. Even for some cases that EEG could not detect, fNIRS could detect them. For motor execution, the average classification accuracy by EEG alone was 90%, but EEG + HbR provided 93%, see Table 1 in Fazli et al. (2012). The previous studies on single modality have shown that the classification accuracy for two commands using fNIRS was about 65% (Stangl et al., 2013), and that for four commands using EEG (rotations and movements of the left/right wrists) was about 65% (Vuckovic and Sepulveda, 2012). The objective in this paper, however, is to generate more commands without losing the classification accuracy. In this paper, four commands will be generated: two from the prefrontal cortex and two from the motor cortex by configuring fNIRS and EEG in such a way that each control signal is generated from its associated brain region. In this way, a new command can be generated in every 0.6 s and the achieved classification accuracy was over 80%.

The BCI techniques to control a wheel chair are diverse: eye movement and blinking based (Gneo et al., 2011; Lin and Yang, 2012), emotions based (Fattouh et al., 2013), and event related and state control (Galán et al., 2008; Huang et al., 2012; Carlson and Millán, 2013). All these are reactive BCI, in which output from the brain is generated in reaction to an external stimulation. The novelty in this work is the proposition of a hybrid configuration of EEG and fNIRS for active BCI, whose classification accuracy is over 80%. Using four brain tasks (left/right motor execution, mental counting, and mental arithmetic), four commands were generated. In the proposed configuration, EEG electrodes are placed on the motor cortex region and NIRS optodes on the prefrontal cortex region. The left and right directions were decoded by tapping of the left or the right hand, and the mental arithmetic and the mental counting was used to decode backward and forward directions. The classification accuracies of the 12 subjects justify that the proposed configuration is suiltable for BCI and direction decoding which can be used for the generation of control commands for movement executions for the patients suffering from lower-limb disorders.

## Methods

### Participants and experimental paradigm

**Figure 1:**
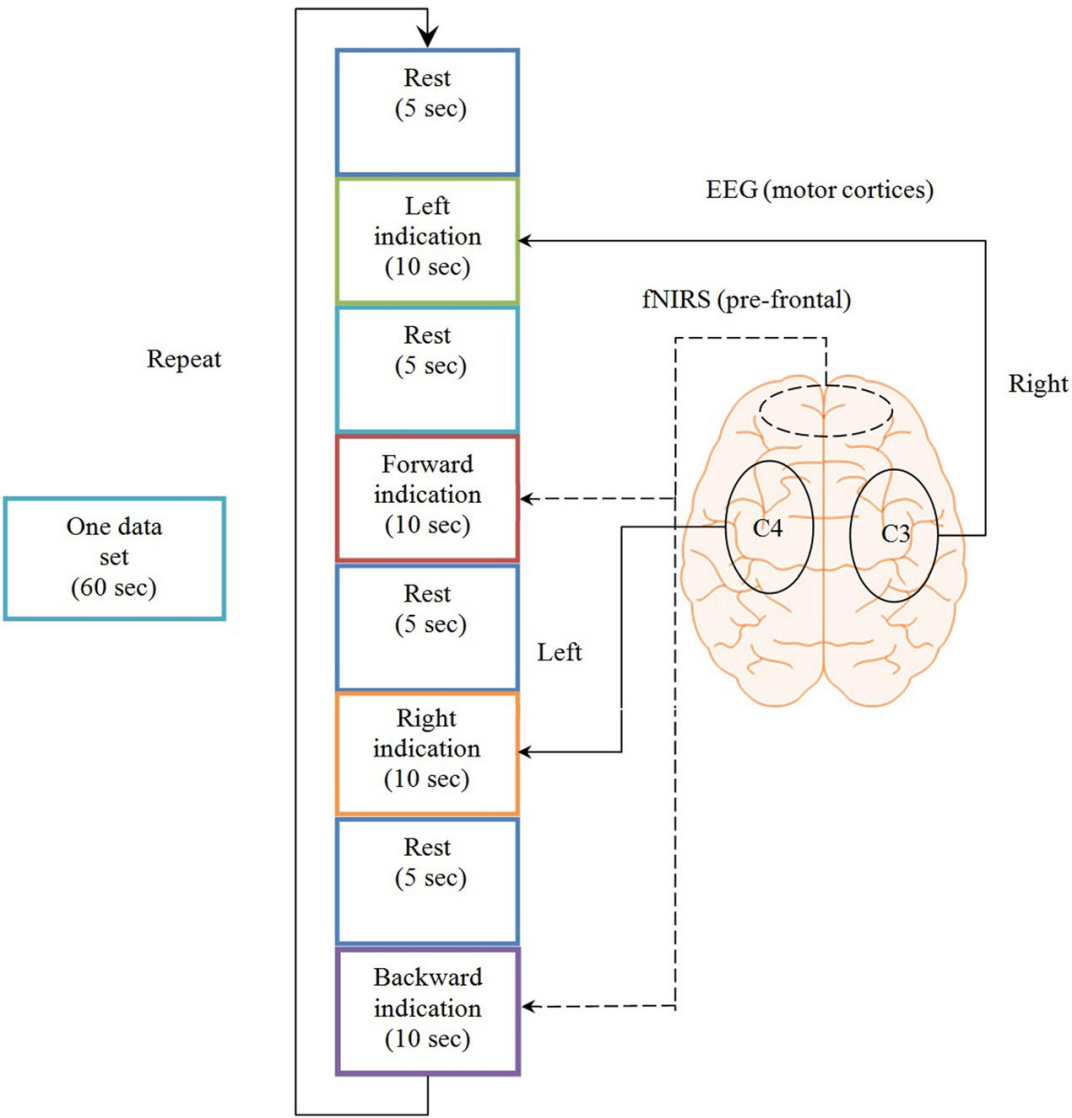
**Experimental paradigm**. One complete data set over the span of a minute, consisting of four rest periods, two task periods detected by NIRS from the prefrontal brain region and two motor execution periods detected from C3 and C4 regions of brain using EEG.

Twelve healthy volunteers (all male;10 right handed, two left handed; aged 24–34 years) participated in the experiment. The experiment was conducted under the declaration of Helsinki and consent was taken from the subjects priror to the start of experiment. The experiment was performed in a confined room to reduce disturbance from the environment. The subjects were sat in a comfortable chair with their arms on arm rests and instructed to relax. A screen nearly 70 cm away from the subjects was placed on which left, right, forward, and backward arrows were displayed. On display of each arrow, a time marker starts at the bottom of screen indicationg the start and end of stimulus. For right and left directions, the subjects were asked to tap their associated hands 10 times during the time period shown on the screen for 10 s. For forward and backward directions, the subjects were asked to do mental counting for 10 s and arithmetic subtraction for 10 s. A training session was performed before the start to make the subjects familier with the paradigm. The total duration of each experiment for each subject was 5 min, divided into rest and activity periods. The time duration of each data sample was 60 s. The initial 5 s of the experiment was the rest period, after which the subjects were shown left or right direction symbols and requested to physically tap their left or right hand accordingly, at a frequency of 1 Hz over a 10 s interval. The subjects were also instructed to increase the strength of tap for left hand for better discrimination of signals. The next 5 s was another rest period, subsequent to which the subjects were shown a forward or backward direction symbol for an interval of 10 s. The subjects were instructed to perform arithmetic subtraction, on a task sheet placed 25 cm away prior to the start of the experiment, upon the display of the backward direction symbol. For the mental arithmetic task, the subjects were asked to mentally perform a series of arithmetic calculations that were given on the sheet in a pseudorandom order. These calculations consisted of subtraction of a two-digit number (between 10 and 20) from a three-digit number throughout the task period with successive subtraction of a two-digit number from the result of the previous subtraction (e.g., 695-19, 706-12, 894-15, etc). This mental activation period was followed by a 5-s rest period, after which the subjects were again shown the left or right direction symbol and instructed, once again, to tap their hand at a 1 Hz frequency over the time span of 10 s. If in a previous case the subjects were shown the left direction symbol, the next time the indicated direction symbol was the opposite one. The directed hand activity was followed by 5 s rest, which was followed in turn by 10 s of mental activation. In this case as well, the next shown direction was reversed, according to which the subjects were asked to perform an arithmetic counting for indicated forward direction. For the mental counting task, the subjects were asked to mentally count down from number “99” backwards. For “Stop” the subjects were asked not to perform any activity. A check on EEG and NIRS data values was placed to distinguish the activation and resting state by defining the baseline and activity. The movement command can only be produced if there is activity in any one of the four brain regions else the state should be termed as “Stop” state. Figure 1 shows one complete sample process; the second sample was taken immediately after the obtainment of the first.

### Sensor configuration

Eight EEG electrodes were placed on the motor cortex region on the scalp and 12 channel NIRS was placed on the prefrontal brain region. The reason for using NIRS on the prefrontal cortex is because it can discriminate between two activities from the prefrontal region with high classification accuracies (Naito et al., 2007; Power et al., 2010; Verner et al., 2013; Naseer et al., 2014) whereas the same cannot be done using EEG (Knyazev, 2013). Meanwhile NIRS signals are affected by dense hairs (Gervain et al., 2011) making EEG a better option for the detection of brain activities from the motor cortex region. Furthermore, if both modalities are positioned at the same brain location, they induce noise in each other thus reducing the strength of obtained signals for BCI (Safaie et al., 2013). Using the current setup, four signals were obtained thus enhancing the performance of NIRS by combining with EEG setup.

### Data acquisition

**Figure 2:**
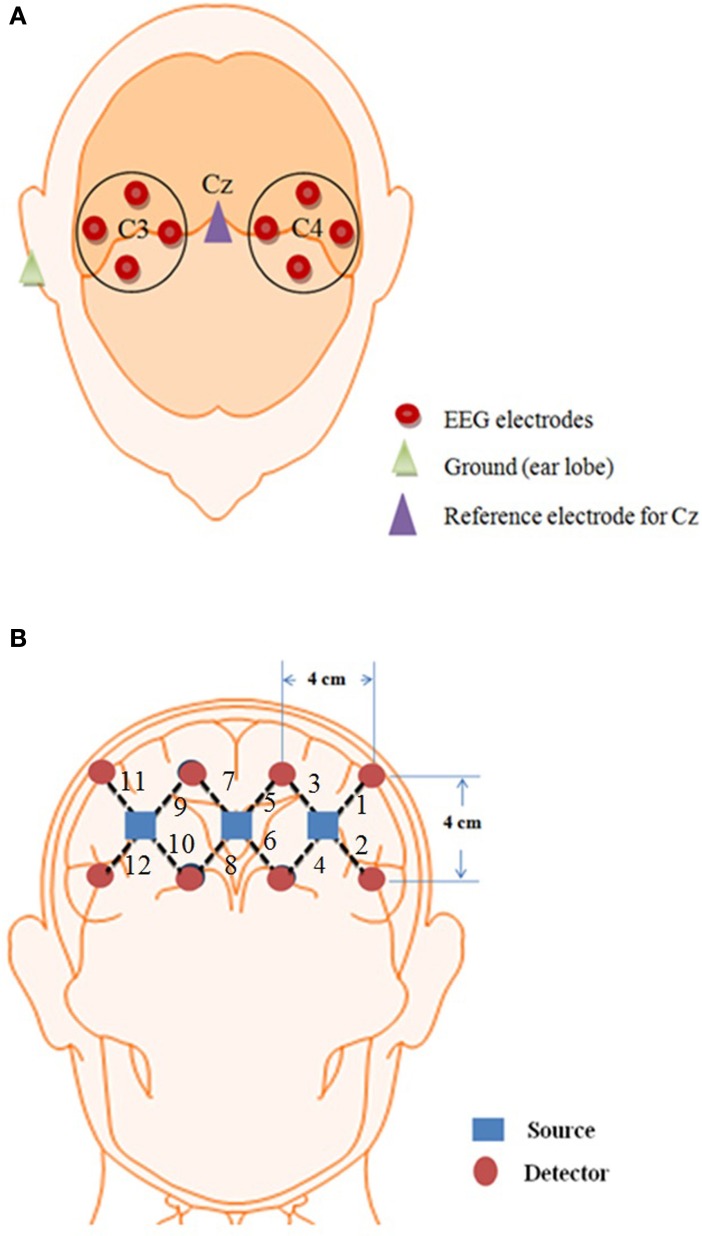
**Optode location for EEG and NIRS. (A)** 8 EEG electrodes placement over the Cz of the brain, **(B)** 12 channel locations on the prefrontal brain region using three sources and eight detectors.

The brain activities related to the mental tasks and motor executions were measured from the NIRS and EEG, respectively. Eight Ag/AgCl EEG electrodes were placed on C3, C4, T3, T4, P3, P4, F3, and F4 locations according to the International 10–20 system (Homan et al., 1987; Pivick et al., 1993; Jurcak et al., 2007), and the data were recorded by g-MOBIlab+ biosignal acquisition device (Christoph Guger, Austria) at a sampling rate of 256 Hz. The NIRS-System (DYNOT, NIRx Medical Technologies, USA) was used in this experiment with wave length of 760 and 830 nm, respectively. A total of three sources and eight detectors forming a combinational pair of 12 channels were used in the experiment. This assembly was placed on Fp1 and Fp2 regions of the brain and the optodes were placed in the way that they cover the whole prefrontal area in order to maximize the probability of locating the activated region of brain. The sampling frequency used for the acquisition of NIRS signals was 1.81 Hz. Figure 2 shows the source-detector locations for the optode placement for EEG and NIRS.

### Data analysis

**Figure 3:**
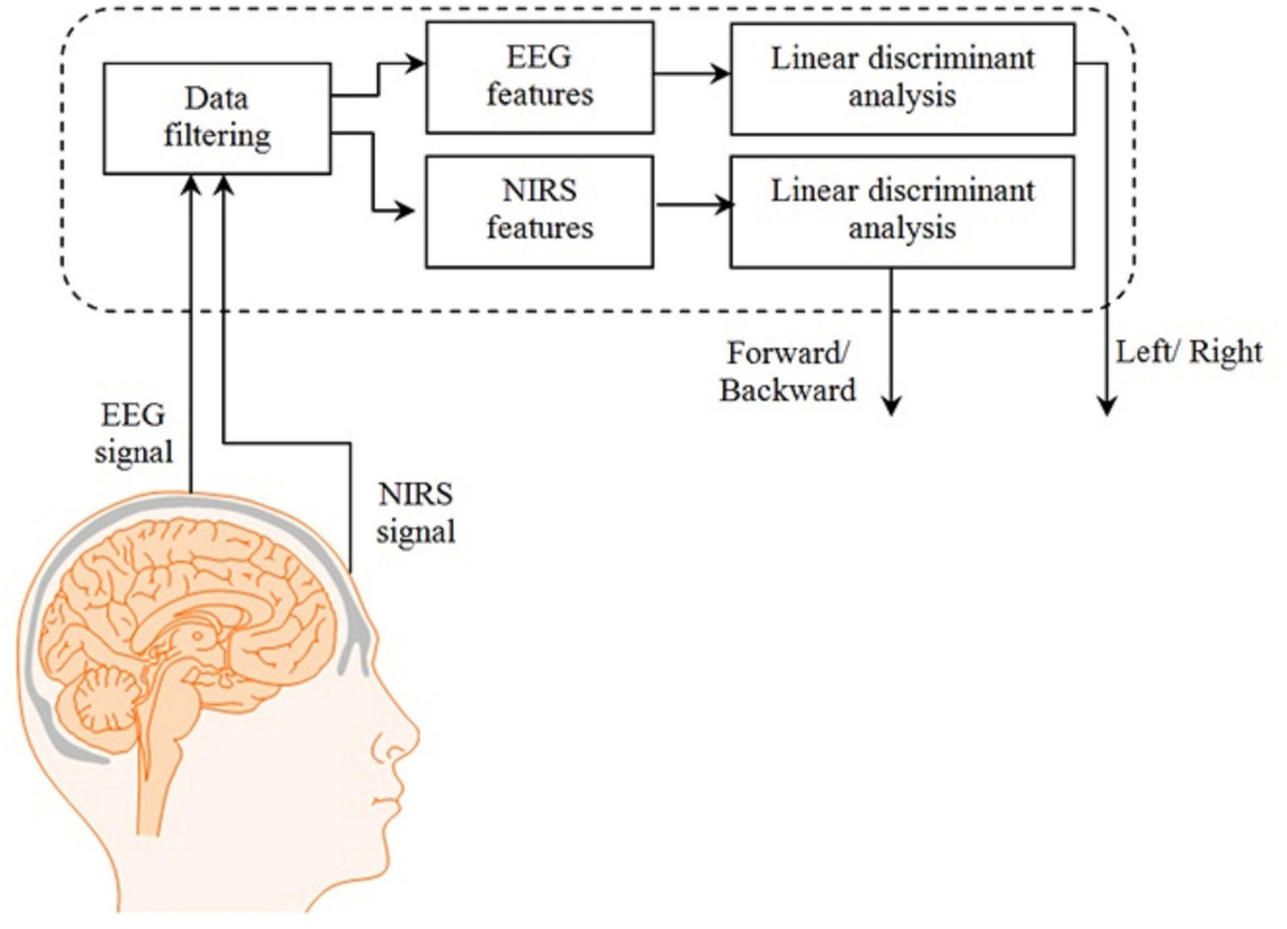
**System block diagram**. The complete process from signal acquisition to control commands generation.

The fNIRS signals were obtained using the modified Beer-Lambert law (Coyle et al., 2007; Hu et al., 2010, 2012; Kamran and Hong, 2013; Naseer and Hong, 2013).

(1) $$A\left(t;\lambda \right)=\ln\frac{I_{in}\left(\lambda \right)}{I_{out}\left(t;\lambda \right)}=\alpha \left(\lambda \right)\times c\left(\lambda \right)\times l\times d\left(\lambda \right)+\eta ,$$

(2) $$\left[\begin{array}{c} \Delta c_{\text{HbO}}(t)\\ \Delta c_{\text{HbR}}(t) \end{array}\right]={\left[\begin{array}{cc} \alpha_{\text{HbO}}\left(\lambda_{1}\right) & \alpha_{\text{HbR}}\left(\lambda_{1}\right)\\ \alpha_{\text{HbO}}\left(\lambda_{2}\right) & \alpha_{\text{HbR}}\left(\lambda_{2}\right) \end{array}\right]}^{-1}\left[\begin{array}{c} \Delta A\left(t;\lambda_{1}\right)\\ \Delta A\left(t;\lambda_{2}\right) \end{array}\right]\cdot \frac{1}{l\times d\text{​}\left(\lambda \right)},$$

where *A* is the absorbance of light (optical density), *I_in_* is the incident intensity of light, *I*_out_ is the detected intensity of light, α is the specific extinction coefficient in μM^−1^cm^−1^, *c* is the absorber concentration in μM, *l* is the distance between the source and detector in cm, *d* is the differential path-length factor, and η is the loss of light due to scattering. In order to remove noise from the hemodynamic response, different techniques are used (Santosa et al., 2013). In the present study, respiration- and pulse-related noises were removed from the data using Gaussian low-pass filtering and wavelet transform (Ye et al., 2009; Hu et al., 2013). The β-band falling within the 12–30 Hz range was obtained by online band-pass filtering of the EEG signals (Zaepffel et al., 2013; Kaiser et al., 2014). The epochs lasting 10 s were estimated by segmenting the recordings from +1 to +11 s relative to the onset of the tapping stimulus, thus yielding five epochs for each left and right hand activity (Delorme and Makeig, 2004; Subasi and Gursoy, 2010; Turnip et al., 2011; Turnip and Hong, 2012). Linear discriminant analysis was used as the classifier. The features in the case of EEG were the mean values of peak amplitudes of channels C3 and C4 whereas in the case of NIRS, the mean values of HbO and HbR were used (Lotte et al., 2007; Zhang et al., 2013; Naseer et al., 2014). An online analysis was performed on the data by downsampling EEG data to 1.82 Hz to synchronize with the NIRS sampling rate. For both modalities, one data sample was obtained approximately after 0.5 s, which was then processed at 250 Hz to obtain the control command. Figure 3 shows the complete process for control command genereation for BCI.

## Results

**Figure 4:**
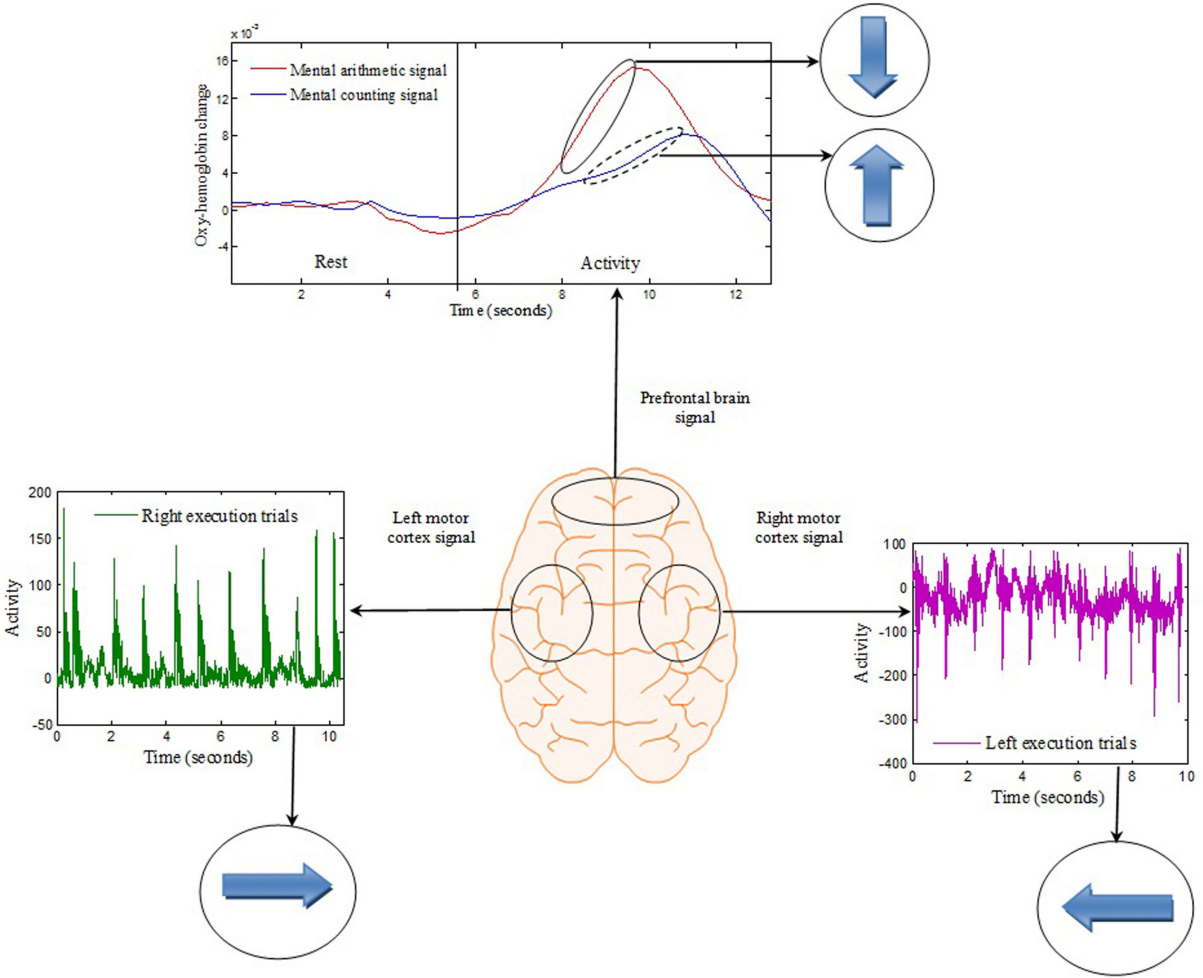
**Brain-signal acquisition:** Each spike from the left or right motor cortex represents a hand tapping with the right or left hand, respectively. The concentration change in the prefrontal brain region reflects the change from rest to mental counting (“forward”) and mental subtraction (“backward”).

The signals extracted from the left and right brain hemispheres (the C3 and C4 regions) and the frontal brain hemisphere (the Fp1 and Fp2 regions) are shown in Figure 4. The signal from the motor cortex region reflects the action potential generated due to the firing of neurons when an motor execution task was performed. The signal from the prefrontal region is the hemodynamic change of HbO from the rest to mental execution period.

**Figure 5:**
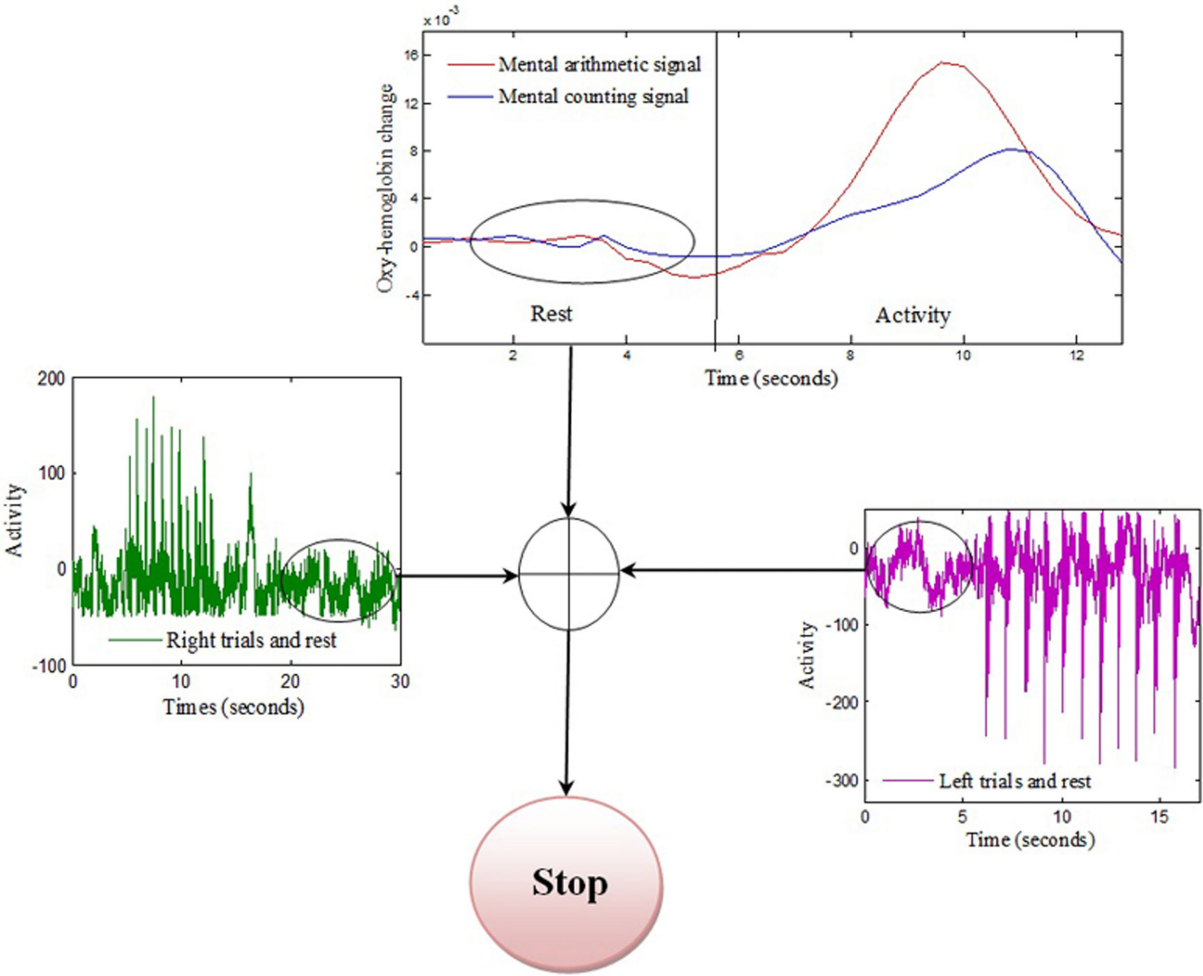
**Stop condition:** Common rest state of the three signals is selected as stop command. The “Stop” command can only be generated if absolute rest is detected from three brain regions.

The association between movement and rest state was developed by taking the common rest state as “Stop” indication. This is also beneficial as using this methodology only one command can be generated at one time and thus reduces the chance of miss-classification. The movement command can only be produced if there is activity in any one of the four brain regions else the state should be termed as “Stop” state as shown in Figure 5 and Table 1.

**Table 1:** **Brain activities of EEG and NIRS for different control commands**.

**Brain activity**		**Command**
**EEG**	**NIRS**	
Left hand tapping	Rest	Left
Right hand tapping	Rest	Right
Rest	Mental arithmetic	Back
Rest	Mental counting	Forward
Rest	Rest	Stop

**Figure 6:**
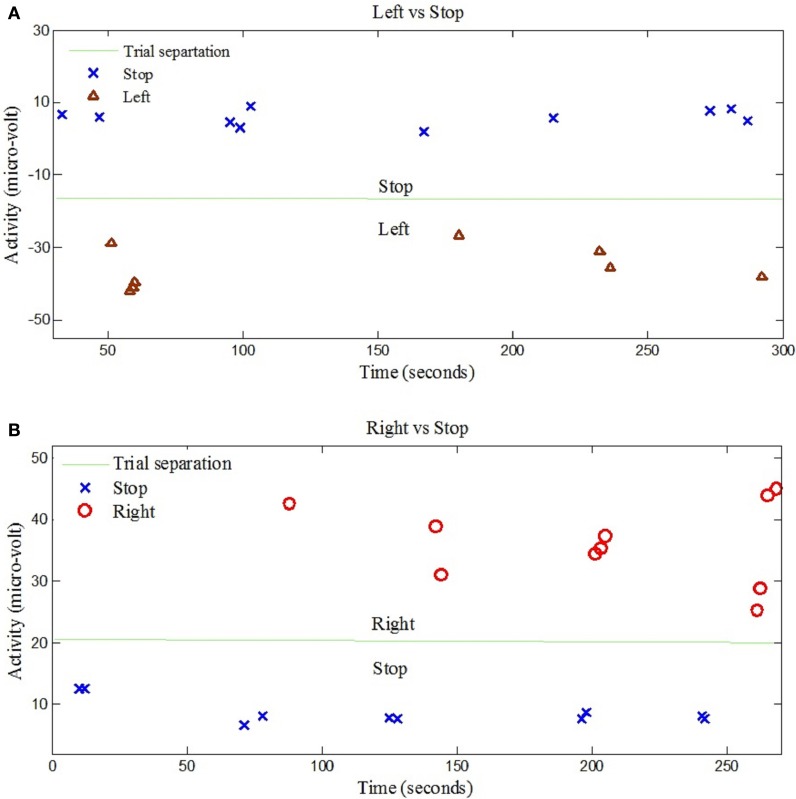
**EEG data classification for left and right control commands. (A)** Shows the classification of trials for “Left” and “Stop” command signals for Subject 7, **(B)** Shows the classification of trials for “Right” and “Stop” command signals for Subject 6.

**Figure 7:**
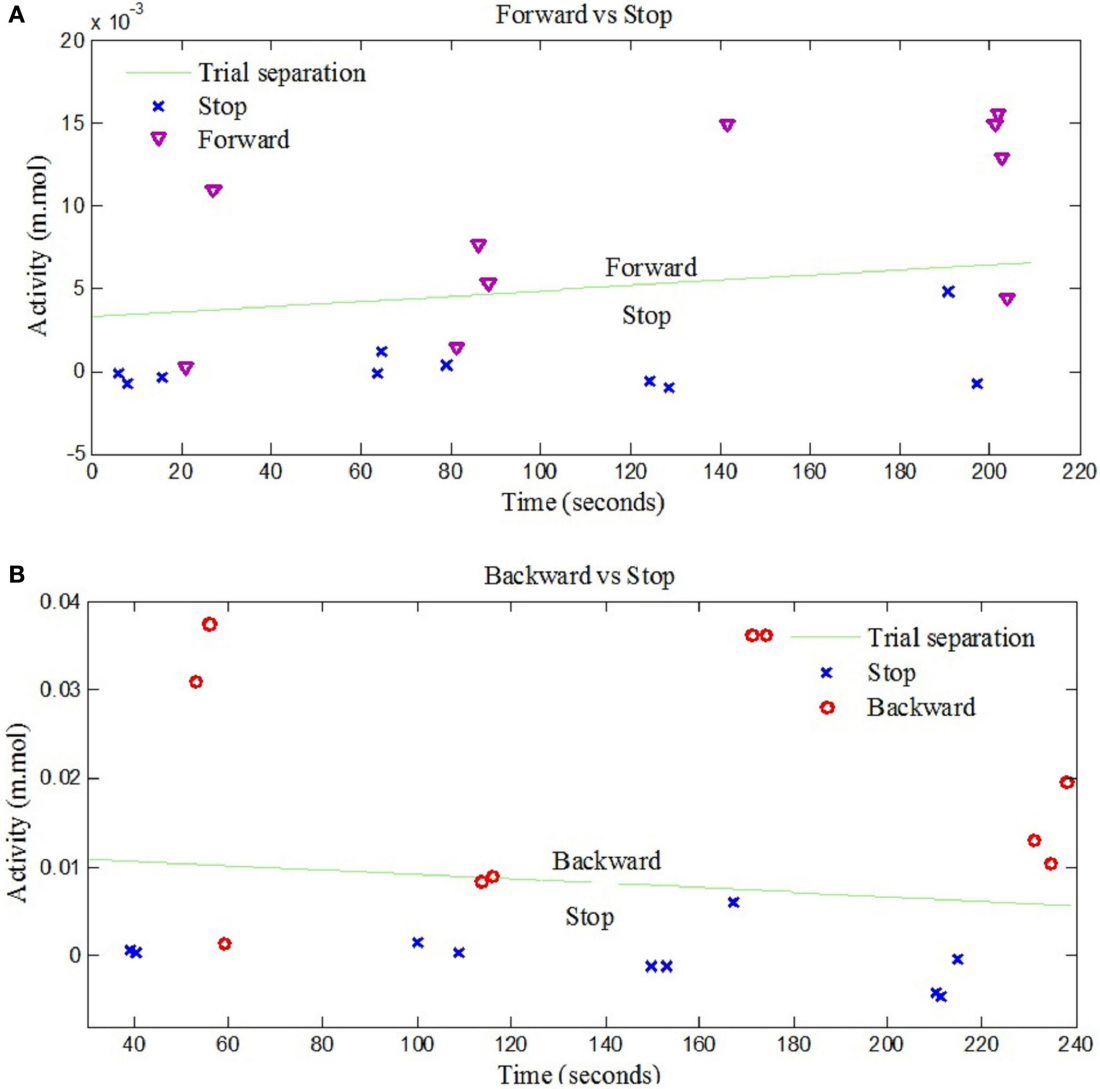
**NIRS data classification for forward and backward control commands. (A)** Shows the classification of trials for “Forward” and “Stop” command signals for Subject 3, **(B)** Shows the classification of trials for “Backward” and “Stop” command signals for Subject 7.

The left- and right-signal classifications are shown in Figure 6. Similary forward and backward mental tasks are represented in Figure 7. Figures 5, 6 show changes of state of data with respect to time. The results indicate a significant difference between the states. Table 2 lists the classification accuracies for all trials of the 12 subjects.

**Table 2:** **Classification accuracies: four control command accuracies of 12 subjects for left vs. stop, right vs. stop, forward vs. stop, and backward vs. stop**.

**Subject**	**Left vs. Stop (%)**	**Right vs. Stop (%)**	**Forward vs. Stop (%)**	**Back vs. Stop (%)**
1	98.4	98.7	81.1	86.5
2	99.8	99.2	79.1	84.1
3	93.2	94.7	81.2	85.4
4	97.7	96.5	80.1	80.8
5	98.1	88.2	81.2	86.9
6	91.1	99.5	84.2	86.6
7	99.2	98.1	77.2	81.1
8	98.0	98.2	83.1	87.1
9	91.2	90.4	80.2	83.1
10	85.0	84.1	74.1	76.3
11	94.5	95.2	82.8	86.1
12	90.1	94.0	78.2	79.3
Mean	94.7 ± 4.6	94.7 ± 4.8	80.2 ± 2.7	83.6 ± 3.5

## Discussion

The first objective of this study, which was achieved, was to detect four different classes of signal for generation of control commands for movement estimation suitable for BCI purposes. Two classes of signal were obtained by EEG using motor execution from the left and right motor cortex regions, and two classes are obtained by NIRS from the prefrontal brain region using mental counting and arithmetic. These two tasks are simple tasks that are very easy to perform and can be detected from the prefrontal cortex. For the decoding application they serve their purpose well and have already been used in several BCI studies (Naito et al., 2007; Power et al., 2012; Naseer et al., 2014). Figure 4 clearly shows that each tap recorded from the left and right brain hemispheres due to the motor execution was recorded as a signal spike from the reference position. The time interval between each tap was maintained consistent at approximately 1 s. In order to differentiate between the left- and right-executed signals, each trial was separated by a time interval of 20 s. It was observed that the HbO concentration changes during the mental task began to increase 2 s after the subjects were prompted, and that the HbO level required almost 12 s to settle after the termination of that signal. Accordingly, the time gap between the two NIRS tasks was set at 20 s.

The classification of left and right hand tapping is performed by increasing the magnitude of left hand tapping from the right hand tapping. It has been reported in Jochumsen et al. (2013) and Robinson et al. (2013) that the neuronal response due to fast and slow movement is different and can be distinguished with high classification accuracy. Figures 6, 7 show clearly that the four corresponding signals are separable and, thus, utilizable for generation of BCI control commands.

The observed classification accuracies for the 12 subjects significantly show that the proposed research is suitable for BCI purpose. In comparison to other studies in which two control commands are generated based on motor and arithmetic tasks with low classification accuracies results (Stangl et al., 2013), the results have shown potential for control command generation with high classification accuracy. Moreover four stage classifier is required if only one modality is used for four signals acquisition which may results in significant decrease in accuracy (Vuckovic and Sepulveda, 2012). Using the current setup, the same results with better accuracy are achieved: The accuracies of “Forward vs. Stop” and “Backward vs. Stop” trials were 80.2% and 83.6%, respectively, due to the selection of oxy-hemoglobin (HbO) as a classification feature for both control commands. This is the first time that four intended movements are decoded using a hybrid BCI. Wolpaw and McFarland (2004) and the previous two hybrid EEG-NIRS studies (Fazli et al., 2012; Kaiser et al., 2014) have shown discrimination of two signals that can be used to generate two control commands whereas in this paper four control commands have been generated.

The generated commands can be used for those people who cannot perform motor imagery (Vidaurre and Blankertz, 2010) or in such cases that the patient cannot touch any machine directly, for instance, prosthetic legs, working with remote controlled devices, etc. Naseer et al. (2014) have shown binary decision decoding for rehabilitation, whereas in this research the four control commands can be associated to four different decisions. Moreover, the previous hybrid EEG based researches for rehabilitation have shown the use of P300 and steady state visual evoked potentials (Li et al., 2013; Xu et al., 2013) to generate four control commands based on reactive tasks. As motor imagery and motor execution activate the same brain area (Beisteiner et al., 1995; Porro et al., 1996), the same goal can be achived by this research using active tasks. Also, it is known that fNIRS can decode multiple signals from the same brain area: for instance, mental counting and music imagery (Power et al., 2012) and picture imagery and mental arithmetic (Naito et al., 2007) from the same prefrontal cortex. Therefore, there is a potential that the total number of commands in the current EEG and fNIRS configuration can be increased to up to seven. Further research can be carried out using advanced adaptive filtering techniques (Rehan and Hong, 2013), optimal feature sets, and/or combined EEG-NIRS features to achieve better classification results that can be used by patients with lower limb disorder to control wheelchair or prostheses.

## Conclusion

In this research, a hybrid fNIRS and EEG configuration for decoding four movment commands was proposed. The NIRS setup was used to decode the prefrontal activities based on hemodynamic changes of HbO using mental arithmetic and mental counting as tasks. The EEG response detected from the motor cortex region was used to decode other two direction commands. Both modalities were synchronized to obtain control commands at the same time. The results of classification accuracies were highly encouraging and certainly will prove fruitfully applicable to BCI systems and purposes. The use of wirelss systems and translation of the control commands into machine codes will enable effective control of a robotic system suitable for rehabilitation purposes.

## Author contributions

M. Jawad Khan has conducted all the experiments and carried out the data processing. Melissa J. Hong has examined the data and participated in revising the manuscript. Keum-Shik Hong has suggested the theoretical aspects of the current study and supervised all the process from the beginning. All the authors have approved the final manuscript.

### Conflict of interest statement

The authors declare that the research was conducted in the absence of any commercial or financial relationships that could be construed as a potential conflict of interest.
